# Platelet-rich fibrin improves the osteoneogenesis in non-critical defects in calvaria: a histological and histometric study

**DOI:** 10.1590/acb383423

**Published:** 2023-10-13

**Authors:** Evans Soares de Oliveira, Jurandir Marcondes Ribas-Filho, Marcos Sigwalt, Elora Sampaio Lourenço, Fernanda Piraja Figueiredo, Nicolau Gregori Czeczko, Allan Fernando Giovanini

**Affiliations:** 1 Faculdade Evangélica do Paraná – Program in Principles of Surgery – Intituto de Pesquisas Médicas – Curitiba (Paraná) – Brazil.; 2 Universidade Presbiteriana Mackenzie – São Paulo (São Paulo) – Brazil.

**Keywords:** Platelet-Rich Fibrin, Bone Regeneration, Rabbits

## Abstract

**Purpose:**

The aim of this study was to evaluate the effect of platelet-rich fibrin (PRF) and autograft on non-critical bone repair.

**Methods:**

Four bone defects (8.3 × 2 mm) were produced on the calvarium of 15 rabbits. The surgical defects were treated with either autograft, autograft associated to PRF, PRF alone, and sham. Animals were euthanized on the second, fourth or sixth posteoperative week. Histological analyses for presence of bone development on deffect was evaluated comparing the groups treated with autograft and without the autograft separately within the same period. Mann-Whitney’s tests were used to compare the percentage of bone repair in each post-operative period for autograft × autograft + PRF groups and also for control × PRF groups (α = 5%).

**Results:**

No differences were observed between the groups that received autograft and autograft associated to PRF on the second and fourth postoperative week, but areas treated with PRF demonstrated significant osteogenesis when compared to sham group on the fourth and sixth weeks. The groups that received PRF (with autograft or alone) demonstrated an enlarged bone deposition when compared to their control group.

**Conclusions:**

The use of PRF may influence bone repair and improve the bone deposition in late period of repair demonstrating osteoconductive and osteogenic properties.

## Introduction

The development of biological surgical adjuvants associated to autgraft for the local stimulation of bone healing remains an important field of research in tissue engeneering^1^. An ideal supplementar biomaterial should have several characterstics that include ostegenic and osteoconductor properties; not induce an immune response from the host; stimulate angiogenesis; and be completely replaced by bone in quantity and quality similar to that of the host^2^.

In this perspective, the platelet-rich plasma (PRP) is a nontoxic and nonantigenic autogenous blood-derived portion comprising high amounts of leukocytes and platelets. PRP may be responsible for the synthesis and increase of expression of growth factors, increase the osteoblast proliferation and extra cellular matrix synthesis, relevant conditions to promote bone healing^3^,^4^. Since Marx demonstrated a larger and faster new bone formation in artificial bone defects when using a platelet concentrate, it became a promising and important alternative for the treatment craniofacial bone defects for its easy application in clinical practice and beneficial results^5^.

Despite these attractive biological properties, an improvement over the usual PRP was proposed adding fibrin in its composition. The PRF mimics an autologous cicatricial matrix that adds the fibrin glue and a classical platelet concentrate^6^,^7^. PRF consists of a fibrin matrix polymerized in a tetramolecular structure, with incorporation of platelets, leucocytes, cytokines, and hematopoietic stem cells, that may improve the bone regeneration^8^,^9^.

Thus, the aim of this study was to evaluate the bone repair of non-critical defects in rabbit calvaria using PRF alone and in association with particulate autogenous bone to evaluate their osteoconductive and osteoinductive effects.

## Methods

Fifteen white New Zealand female rabbits (140 to 170 days old; 2,456 – 3,502 g) with no previous disease were used following a protocol approved by the Institutional Animal Care and Use Committee (under protocol no. 1896/2017). The rabbits were kept in a room with controlled temperature (~20 to 23°C) and maintained under a 12 h light-dark cycle.

### Surgical procedure

The animals were anesthetized with 5% intramuscular ketamine hydrochloride at a rate of 60 mg/kg associated with xylazine at a rate of 10 mg/kg. Anesthesia was considered effective when the animal was immobile during handling.

The surgical area was previously shaved and asseptically prepared. Sterile barriers limited surgical field. The region was injected sub-periosteally with 1 mL of lidocaine 2% with adrenaline 1:100,000. A midline dermo-periosteal incision (5 cm) was made, raising a skin-periosteal flap to expose the calvarial surface. Four defects of 8.3 mm in diameter were created with a trephine under profuse saline solution irrigation. Autogenous bone fragments were particled for autgraft. The defects were filled:

- With autogenous bone from the bone particulate;
- With particulate autogenous bone associated with PRF;
- Only with PRF;
- No grafted material (as a control = sham).

Posteriorly, the wound was closed using 4 mononylon thread. Afterwards, the region was disinfected again with povidone-iodine (PVPI), and the animals were transferred to the same preoperative conditions. Antibiotic prophylaxis was continued for three days.

### Platelet-rich fibrin fabrication

Eight mililliters of blood were collected on the external jugular vein of each animal and were immediately centrifugeg (Monteserrat model 80-2B) at 1,800 rpm for 10 min (RCF-clot = 182 g). PRF membranes were produced using an Intraspin centrifugation device (45° rotor angulation, 50-mm radius at the clot, 80 mm at the maximum using 10-mL glass-coated polipropilene tubes).

No additives were used in the vacuum tubes. After centrifugation, it was possible to recognize the PRF distinguished from the clot. The clot was detached and removed using sterile tweezers. The PRF was then removed from the bottom of the tube using sterile tweezers and immediately applied in the surgical bed, alone or mixed to autgraft.

### Tissue processing

The animals were euthanized using intravenous administration of pentobarbital 100 mg/kg intravenously after two, four and six postoperative weeks (five animals in each group). Each calvarium was exposed, and each specimen was properly cut with a piezoelectric motor with an ultrasonic surgical tip model H-SG1 in surgery programming at 150% power and with abundant irrigation, where initially the area of each of the groups was delimited. The aim of the piezoelectric ultrasound was to obtain a thin and accurated bone cut, mantaining the cellular architecture intact. Each fragment of calvaria specimens was fixed in 10% buffered formalin for two days, and then decalcified in 20% formic acid solution (20 mL of formic acid PA, 60 mL of distilled water, and 20 mL of 36% formalin) for one week and sodium citrate for five days.

The specimens were washed with tap water, dehydrated with ascending concentrations of ethanol, cleared in xylene, and embedded in paraffin. Serial sections (3 μm) were cut from each specimen using a microtome (RM2155, Leica Microsystems GmbH, Nussloch, Germany) and stained with Masson’s trichrome to detect the bone reapir area.

**Figure 1:**
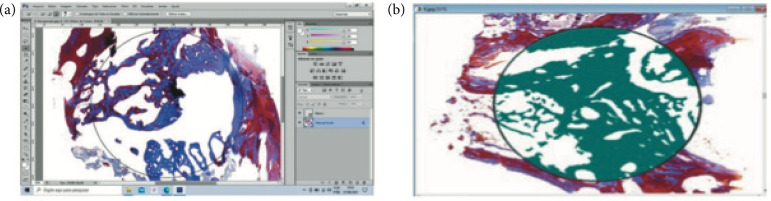
Image Pro Plus program showing: **(a)** bone slide with no-bone tissue removed; **(b)** measurement of the bone area in the circle.

Source: Elaborated by the authors.

From each specimen, 64 slides were obtained. They were scanned in an appropriate histological sacanner to obtain the histological pictures. Each micrograph was opened in the Adobe Photoshop CS6 software, and a circle of 6,027 μm in diameter was drawn from the center of the defect where all the measurements were performed. Using the magic wand tool, all extraosseous tissue was removed from the scanned photo (Fig. 1). In this way, only the bone tissue remained inside the selected area and was mensured. All the measurements were performed by the same operator and saved in jpeg format.

The micrographs exhibiting the interest area were exported to the Image Pro Plus software version 4.5 (Media Cybernetics, United States of America) to obtain the total bone area and the percentage of bone formation in each slide.

### Statistical analysis

Bone percentage was calculated in each slide. The Shapiro-Wilk’s analysis was used to determine the normality of the data. Mann-Whitney’s test was used to compare the percentage of bone repair in each post-operative period for autograft × autograft + PRF groups and also for control × PRF groups. P < 0.05 was considered to be statistically significant.

## Results

### Histomorphometric evaluation

**Figure 2:**
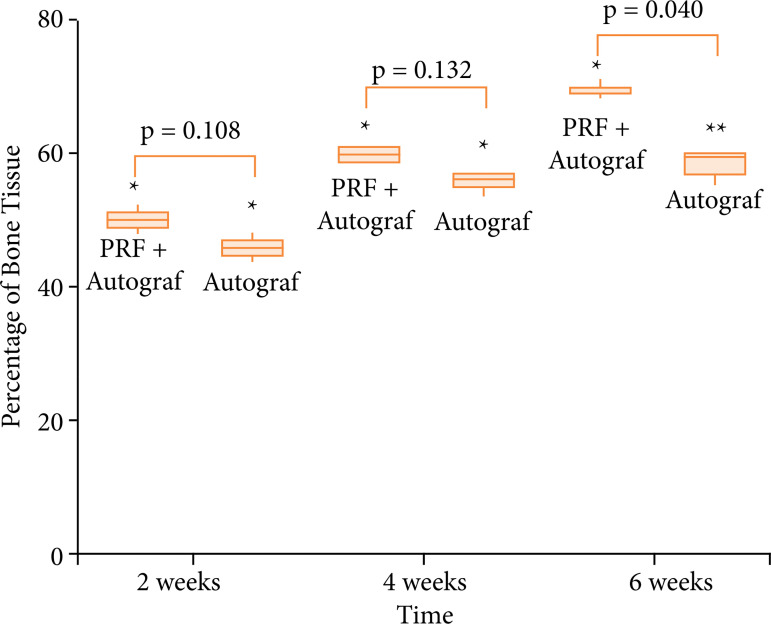
Box-plot graph showing the percentage of bone tissue in the evaluated postoperative times for groups autograft and autograft + PRF.

Source: Elaborated by the authors. PRF: platelet-rich fibrin; *values statistically similar, p > 0.05.

**Figure 3:**
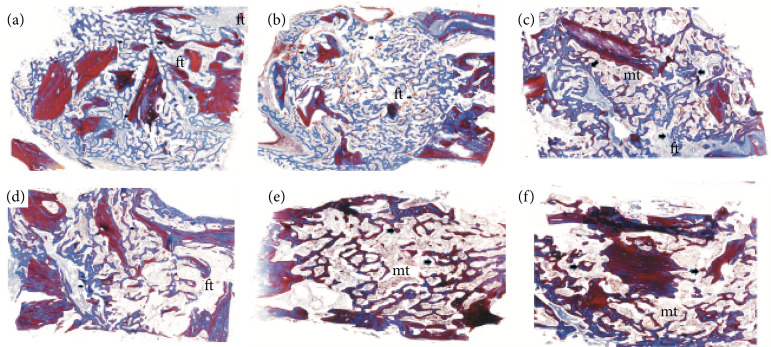
Histological illustration of autograft and autograft mixed to PRF groups demonstrating the quantity of bone formation (arrows) among fibrous tissue (ft) and medullary tissue (mt) on the **(a** and **b)** second week, **(c** and **d)** fourth week, and **(e** and **f)** sixth week post-surgery. Notice the larger bone tissue deposition in autograft PRF when compared to autograft group. (Original magnification 40x).

Source: Elaborated by the authors. PRF: platelet-rich fibrin.

A brief description of the histological findings for each group and post-operative time is summarized ahead. Figure 2 shows the histomorphometric data represented as a box-plot graph, comparing the percentage of bone repair in each post-operative time for the autograft × autograft + PRF groups. Figure 3 presents the histological illustration of autograft and autograft mixed to PRF groups.

**Figure 4:**
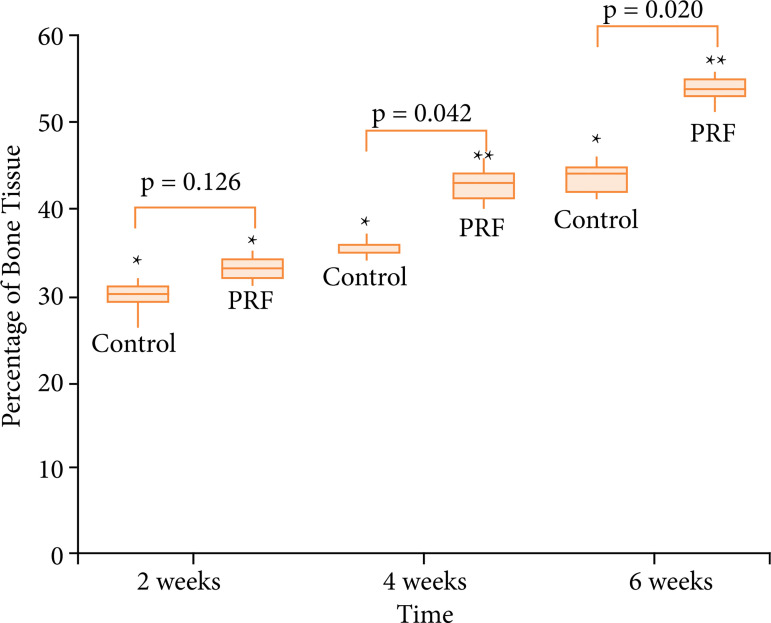
Box-plot graph showing the percentage of bone tissue in the evaluated postoperative times for groups control and PRF.

Source: Elaborated by the authors. PRF: platelet-rich fibrin; *values statistically similar, p > 0.05.

**Figure 5:**
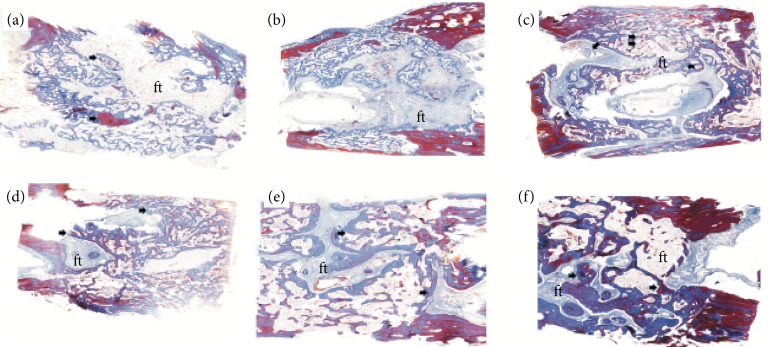
Histological illustration of control and PRF groups demonstrating the quantity of bone formation (arrows) among fibrous tissue (ft) on (**a** and **b**) the second week, (**c** and **d**) the fourth week, and (**e** and **f**) the sixth week post-surgery. Notice the larger bone tissue deposition in PRF when compared to control group. (Original magnification 40x).

Source: Elaborated by the authors. PRF: platelet-rich fibrin; *values statistically similar, p > 0.05.

- Autograft (Fig. 3): on the second week after surgery, it was observed thin and exuberant bone trabecules in the bed of the bone defect surronding the autograft. It was notorious the presence of fibrous among the new bone formed tissues (Fig. 3a). On the fourth week, the microscopic aspects of the regenerative tissue was similar, but an increase in the amount of bone tissue was noted (Fig. 3c). After six weeks, the presence of a cancellous bone with prominent medullary tissue was found in the defects. The increase in bone tissue was slight when compared to the fourth week of analysis. During this period, the presence of cancellous bone tissue surrounded by exuberant and well-formed medullary adipose tissue was evident (Fig. 3e);
- Autograft mixed to PRF (Fig. 3): on the second (Fig. 3b) and fourth (Fig. 3d) postoperative weeks, the histological aspects of bone repair where the PRF was applied were similar to treatment using only autograft. On the sixth week, the bone area demonstrated exhuberant islets of compact and robust Haversian bone among a well-formed medullary tissue (Fig. 3f);

Figure 4 presents the percentage of bone repair in each post-operative time for control × PRF groups. Figure 5 shows the histological illustration of control and PRF groups, as described below.

- Control (sham) (Fig. 5): the histological analysis after two weeks demonstrated an intense amount of connective tissue, with a tenous and delicate deposition of collagen surrounding thin neoformed bone trabeculae (Fig. 5a). The presence of medullary tissue was not observed in this group. After four (Fig. 5c) and six (Fig. 5e) weeks, the histological pattern of repair was similar, with either collagen density or new bone formation increasing with time;

PRF (Fig. 5): the microscopic aspects in this group demonstrated similar regenerative pattern to that of control group in all time periods. However, it was notorious that both collagen density and amount of cancelous bone trabecular tissue were larger when compared to sham group.

## Discussion

This study evaluated the effect of PRF alone and mixed with autograft in the bone repair of non-critical defects in rabbit calvaria using histological and histomorphometrical analysis on the second, fourth and sixth week post-surgery. The use of PRP revealed local increases of bone volume, especially after six weeks. The increase in bone deposition in specimens in which PRF was inserted occurred alone or associated with an autograft, suggesting that PRF induces osteoneogenesis. It has been reported that the effects of PRF seems to be related to its composition, creating a microenvironment that support the osteoneogenesis^6^,^10^,^11^.

A fundamental variable contained in PRF that should be considered is the presence of platelets that are five to ten times higher compared to blood clot^12^,^13^. This large number of platelets release significant quantities of growth factors that increase the rate of angiogenesis and improves the osteodifferentiation in the wound healing area. Corroborating to this premisse, the synthesis and secretion of transforming growth factor-β (TGF-β) seems to be a fundamental cytokine synthesized by platelets during the angiogenesis and osteogenesis^14^. The autocrine and paracrine cellular stimulation by TGF-β produces the expansion of the mesenchymal progenitor cells into the osteoblasts progenitors^15^,^16^. Thus, the TGF-β also promotes osteoprogenitor proliferation, early differentiation, and commitment to the osteoblastic lineage through the selective mitogen-activated protein kinases (MAPKs) and Smad2/3 pathways, and the cooperation between TGF-β with Wnt and BMP that are responsible for osteoblast differentiation^17^.

Associated to secretion of TGF-β, platelets also produce insulin-like growth factor-1 (IGF-1) from its α granules upon activation^18^, which is an important growth factor that activates RUNX-2^19^, improving not only the osteogenenic expression, but also the phosphatase alkaline and mineral deposition in regenerative sites^20^. It is noteworthy that when the IGF-1 phosphorylates is a receptor in preosteoblast stem cell, this biological action produces intracellular activation of substrate insulin receptor substrate-1 (IRS-1), that, in turn, is responsible for Akt activation, which contributes to the survival signal of IGF-1^21^ and consequently the increase of osteoprotein RUNX-2 for osteoconduction and osteoinduction^6^.

On the other hand, the presence of fibrin seems to be crucial during the repair. Its presence creates micropores composed of thin fibrin fibers form within clots and may function as scaffolds for cell adhesion, as well as for the delivery of growth factors produced by platelets^22^.

It should be highligthted that the gradual release of growth factors stored in fibrin to their surrounding tissues may be considered important, since it prolongs the growth factor activity that is ideal for tissue engineering and may be associated to success of PRF, specially when compared to PRP, that in several study not only failed to promote osteogenesis, but also produced a pathological microenvironment during the bone repair, as demonstrated by our group^3^,^14^,^23^.

Despite the numerous intrinsic effects of PRF that are considered beneficial for osteogenesis, the results during the early periods of bone repair did not demonstrate a significant increase in osteogenesis when compared to the control or autograft group. These results differ from previous studies that demonstrated a robust increase in osteogenic activity when PRF was used^24^. These differences in results may be attributed to PRF fabrication^25^.

Thus, a factor that may contribute to the lack of robust osteogenesis in early repair periods may be attributed to the type of tube used in centrifugation^26^. In this context, Tsujino et al.^27^ demonstrated that PRF centrifuged in tubes containing silica produces incorporation and contamination by this component among the PFR net of fibrin. The deleterious presence of silica was demonstrated by Masuki et al.^28^, who verified that the silica microparticles were adsorbed on periostal cell surface and, as a result of the interaction, induced apoptosis of periostal cells, with a significant reduction in cell proliferation and viability, as well as platelets disruption.

In addition, Miron et al.^29^ demonstrated that PRF membranes produced with glass tubes were larger (~200%) than those produced with plastic tubes regardless the differences in the centrifugation used. These effects combined seem to indicate that PRF tubes have a greater effect on the results and may explain, in part, the lack of exhuberant osteogenesis in earlier stage of repair in the present study.

The manufacture of PRF has undergone intense changes in recent years to improve its osteogenic capacity. Factors such as the speed of revolution per minute and the g force applied to the tube may alter the effects of PRF on osteogenesis. Each of the abovementioned parameters may influence regeneration success with PRF. It has been previously described that PRF clots fabricated at lower centrifugation speeds improve growth factor release and cellular behavior owing to higher cellular content and growth factor accumulation. In this way, PRF fabrication protocols should be described in details as these parameters influence osteogenesis^25^.

## Conclusion

This study demonstrated that the use of PRF increased the bone repair in non-critical defects created in rabbit calvaria when used alone or associated to autograft.

## Data Availability

The data will be available upon request.
